# HIV Incidence in a Cohort of Women at Higher Risk in Beira, Mozambique: Prospective Study 2009–2012

**DOI:** 10.1371/journal.pone.0084979

**Published:** 2014-01-27

**Authors:** Karine Dubé, Arlinda Zango, Janneke van de Wijgert, Ivete Meque, Josefo J. Ferro, Fidelina Cumbe, Pai Lien Chen, Sabrina Ma, Erik Jolles, Afonso Fumo, Merlin L. Robb, Paul J. Feldblum

**Affiliations:** 1 FHI 360, Durham, North Carolina, United States of America; 2 United States Military HIV Research Program (MHRP), Henry M. Jackson Foundation for the Advancement of Military Medicine, Inc. (HJF), Bethesda, Maryland, United States of America; 3 Universidade Católica de Moçambique/Catholic University of Mozambique, (UCM) Centro de Investigação de Doenças Infecciosas/Research Center for Infectious Diseases (CIDI), Beira, Mozambique; 4 Amsterdam Institute for Global Health and Development (AIGHD) and Academic Medical Center of the University of Amsterdam, Amsterdam, The Netherlands; 5 University of Liverpool, Institute of Infection and Global Health, Liverpool, United Kingdom; Public Health Agency of Barcelona, Spain

## Abstract

**Background:**

HIV is prevalent in Sofala Province, Mozambique. To inform future prevention research, we undertook a study in the provincial capital (Beira) to measure HIV incidence in women at higher risk of HIV and assess the feasibility of recruiting and retaining them as research participants.

**Methods:**

Women age 18–35 were recruited from schools and places where women typically meet potential sexual partners. Eligibility criteria included HIV-seronegative status and self-report of at least 2 sexual partners in the last month. History of injection drug use was an exclusion criterion, but pregnancy was not. Participants were scheduled for monthly follow-up for 12 months, when they underwent face-to-face interviews, HIV counseling and testing, and pregnancy testing.

**Results:**

387 women were eligible and contributed follow-up data. Most were from 18–24 years old (median 21). Around one-third of participants (33.8%) reported at least one new sexual partner in the last month. Most women (65.5%) reported not using a modern method of contraception at baseline. Twenty-two women seroconverted for a prospective HIV incidence of 6.5 per 100 woman-years (WY; 95% confidence interval (CI): 4.1–9.9). Factors associated with HIV seroconversion in the multivariable analysis were: number of vaginal sex acts without using condoms with partners besides primary partner in the last 7 days (hazard ratio (HR) 1.7; 95% CI: 1.2–2.5) and using a form of contraception at baseline other than hormonal or condoms (vs. no method; HR 25.3; 95% CI: 2.5–253.5). The overall retention rate was 80.0% for the entire follow-up period.

**Conclusions:**

We found a high HIV incidence in a cohort of young women reporting risky sexual behavior in Beira, Mozambique. HIV prevention programs should be strengthened. Regular HIV testing and condom use should be encouraged, particularly among younger women with multiple sexual partners.

## Introduction

Mozambique, home to roughly 24 million people [1], is one of the Southern African countries hardest hit by the HIV/AIDS epidemic [2]. The first case of HIV/AIDS was reported in 1986 [3], and there are now approximately 1.4 million Mozambicans living with HIV/AIDS [4]. The national adult HIV/AIDS prevalence is 11.5% [4], with the majority of new HIV infections acquired through heterosexual contact.

Mozambique's high HIV prevalence rates first emerged in the central region, along the main transport corridor of Beira [5]. Beira is the capital of central Sofala Province and is Mozambique's second largest city with around 546,000 people (2007 census), on the Indian Ocean with major commercial transit links to Zimbabwe, Zambia and Malawi. HIV rose dramatically in Sofala Province between 1994–1998 and stabilized in 2000 [5]. In 2009 national surveillance, 17.8% of women and 12.6% of men aged 15–49 years old were HIV-infected in Sofala Province, higher than the national gender-specific prevalence rates [4].

Structural factors that facilitated the HIV epidemic in Mozambique included a civil war that lasted from 1980–1992 [6], a legacy of Portuguese colonial rule that fostered a poor health care infrastructure [6], widespread poverty, and proximity to neighboring countries with severe HIV epidemics. Behavioral factors that may have contributed include high mobility/migration, multiple and concurrent partnerships, low levels of condom use, transactional sex and low levels of male circumcision [4].

Although Mozambique ranked third amongst countries with the largest estimated number of new cases of HIV infection in persons aged 15–49 years old during 2001–2007 [7], reliable HIV incidence data remain limited in Mozambique. The HIV epidemic in Sofala province makes the region a potentially suitable locale for HIV prevention research, but preparatory studies are needed to assess feasibility of recruiting and retaining participants and to ascertain HIV incidence.

As part of a site development initiative, we conducted a prospective cohort study of HIV in Beira, Mozambique to inform the design of future HIV prevention trials. We report here the HIV incidence results and risk factors for HIV acquisition in a cohort of women at higher risk of HIV acquisition.

## Methods

### Ethical considerations

The Comité National de Bioética para a Saúde/National Committee for Bioethics (CNBS) in Maputo, Mozambique, the FHI 360 Protection of Human Subjects Committee (PHSC) and the Division of Human Subjects Protection (DHSP) of the Walter Reed Army Institute of Research (WRAIR) reviewed and approved the study protocol and other essential documents. Written informed consent was obtained from all participants prior to initiating study procedures.

### Study design and population

The primary objective of the study was to measure the incidence of HIV infection prospectively. A secondary objective was to assess the site's ability to enroll and retain a cohort of that size for one year. A cross-sectional survey of over 1,000 women served as the screening phase for the prospective study; baseline results have been published elsewhere [8]. Women age 18–35 were recruited from schools and places where women typically meet potential sexual partners. Eligibility criteria included HIV-seronegative status and self-report of at least 2 sexual partners in the last month. History of injection drug use was an exclusion criterion, but current pregnancy was not.

### Recruitment

We conducted an initial community mapping exercise to identify neighborhoods (*bairros*) with the highest density of places considered to be “higher-risk”, such as bars/brothels, streets known for sex work, fishing markets and kiosks where women meet potential sexual partners. With municipal permission, we recruited students in regular high schools and night schools. The recruitment team also collaborated with a local cultural group, *Nfuma Ya Moçambique*, to raise community awareness about HIV/AIDS, interest in the study and knowledge about the study site, the Research Center for Infectious Diseases (CIDI), affiliated with the Catholic University of Mozambique (UCM). The cultural events also addressed possible rumors about the study. At recruitment encounters, community mobilizers (*activistas*) informed potential study candidates about the study using an ethics committee-approved recruitment script, and later gave candidates a recruitment coupon if they expressed interest to join the study. The CIDI research center offered transportation for study candidates' first visit to the center to show its location.

### Data collection and study procedures

Data collection took place between December 2009 and October 2012. All study procedures were conducted at the CIDI research center by trained staff in private rooms. Baseline, demographic, behavioral, and clinical data were obtained at the screening visit, and rapid testing for HIV and pregnancy was done. Seronegative volunteers were enrolled in the prospective phase and scheduled for monthly follow-up for 12 months, when they underwent face-to-face interviews, HIV counseling and testing, and pregnancy testing. Participants received risk-reduction counseling, free condoms, and were given 150 Meticais reimbursement (about USD $5) at each visit. The community *activistas* acted as follow-up officers and tracers throughout the follow-up period. Women found to be HIV-infected and/or pregnant were referred to relevant care in public clinics. Participants with syndromically diagnosed sexually transmitted infections (STIs) were given treatment prescriptions on-site or referred for additional testing and/or care when necessary.

### Laboratory testing

We performed serial rapid HIV testing first with the Determine HIV-1/2 test (Alere Medical Co. Led. Chiba, Japan), followed by Uni-Gold Recombigen HIV test (HIV Trinity BioTech PLC, Bray, Ireland) if the Determine HIV-1/2 test was positive, according to Mozambique's national HIV testing algorithm. We used SD Bioline HIV-1/2 version 3.0 testing (Standard Diagnostics Inc. Kyonggi-do, Korea) and Vironostika HIV-1 ELISA testing (Uni-Form II Plus 0 test, BioMerieux BV, Boxtel, The Netherlands) to resolve discrepant results. We collected blood and stored plasma at −80°C from each participant at each visit, for possible confirmatory testing.

Incident HIV infection was defined as two positive rapid HIV antibody tests or one positive HIV rapid test confirmed by positive ELISA Vironostika test, after negative results at the previous visit. All incident HIV infections were confirmed by backwards testing of stored specimens of seroconvertors using quantitative HIV-1 RNA PCR. Urine pregnancy testing was done using a rapid human chorionic gonadotropin (hCG) test (Healthease Preg n Care, NEOMED IPA, Tzaneen, South Africa).

### Statistical analysis

The prospective cohort study was designed to enroll approximately 400 women and observe at least 380 person-years of follow-up to conclude that the HIV incidence was no less than 2.1 per 100 woman-years (WY) (two-sided α = 0.05) if the observed incidence was 4.2 per 100 WY.

Data were double-entered into a ClinTrial database (Oracle Health Sciences, Redwood Shores, CA, USA) in Beira and transmitted to the FHI 360 server using Citrix 12.3 software.

We summarized baseline variables using descriptive statistics expressed as mean or median for continuous variables and percentages for categorical variables. Time at risk was defined as the time elapsed from the enrollment date until the last follow-up visit (for participants who remained HIV-negative) or the midpoint between the last PCR negative and the first PCR positive test (for HIV seroconverters).

We estimated HIV incidence in the per-protocol population (met all eligibility criteria and had at least one HIV test result during follow-up) using the number of confirmed HIV seroconversions per 100 WY of follow-up. We calculated the 95% confidence interval (CI) of the incidence using exact methods under the assumption that the number of HIV infections follows a Poisson distribution. All factors associated with incident HIV-1 infection at p≤0.10 in bivariable Cox regression models were considered for inclusion in multivariable Cox regression modeling. All data analyses were conducted using SAS version 9.3 (Cary, North Carolina).

## Results

### Study population

411 women were enrolled in the prospective study, of whom 387 contributed follow-up data (Table 1). Most study participants (79.3%) were between 18–24 years old (median age 21). Educational attainment was good, but most participants (72.7%) were unemployed or had part-time employment (22.4%). The majority (81.8%) were unmarried. The median number of reported sexual partners was two; nearly a third of our sample (29.0%) reported having had more than 2 sexual partners in the last month at baseline and around one third of the participants (33.8%) reported having had at least one new sexual partner in the last month. Most women (65.5%) reported not using a modern method of family planning at baseline and only 9.2% of women reported current condom use for contraception at baseline. Around one out of seven participants (13.9%) reported ever exchanging sex for money or goods. Cleansing the inside of the vagina was almost universal (reported by 96.8% of the women) but vaginal insertions to tighten, dry or heat the vagina were less common (reported by 21%; Table 1).

**Table 1:** Baseline characteristics of women enrolled in prospective cohort, Beira, Mozambique.

	Participants with follow-up	Participants without follow-up	P-value^1^
	N(%)	N(%)	
**Enrolled**	387 (94.2)	24 (5.8)	
**Age(Median (IQR))** 2	21 (19–24)	22 (18.5–25)	0.364
**Education**			
None	7 (1.8)	0 (0.0)	0.786
Primary school (grade 1–5)	39 (10.1)	2 (8.3)	
Secondary school (grade 6–9)	214 (55.3)	16 (66.7)	
High school (grade 10–12) or higher	127 (32.8)	6 (25.0)	
**Employment**			
No, unemployed	284 (73.4)	15 (62.5)	0.152
Yes, part-time	83 (21.5)	9 (37.5)	
Yes, full-time	20 (5.2)	0 (0.00)	
**Ever exchanged sex for money or goods**			
No	330(85.3)	20 (83.3)	0.812
Yes	53 (13.7)	4 (16.7)	
Missing	4 (1.0)	0 (0.0)	
**Number of NEW partners in the last 1 month**			
0	257 (66.4)	15 (62.5)	0.611
1	102 (26.4)	6 (25.0)	
2+	28 (7.2)	3 (12.5)	
**Current Birth Control**			
No	255 (65.9)	14 (58.3)	0.084
Pills/injectable/implant^3^^,^ 4	91 (23.5)	4 (16.7)	
Condoms	34 (8.8)	4 (16.7)	
Other	7 (1.8)	2 (8.3)	
**Number of living births (Mean (Median))**	1.4 (1.0)	1.47 (1.0)	0.872
**Sex with IDU,MSM, or sex worker in the last 1 month**			
No	366 (94.6)	24 (100)	0.624
Yes	21 (5.4)	0 (0.0)	
**Frequency of vaginal cleansing inside the vagina per month**			
31+	317 (81.9)	21 (87.5)	1.000
1–30	55 (14.2)	3 (12.5)	
0	13 (3.4)	0 (0.00)	
Missing	2 (0.5)	0 (0.00)	
**Frequency of vaginal insertions per month**			
1+	77 (19.9)	8 (33.3)	0.271
0	307 (79.3)	16 (66.7)	
Missing	3 (0.8)	0 (0.0)	
**Number of vaginal sex without condom in last 7 days with primary partner (median)**	1 (0–3)	0 (0–1)	0.014
**Number of vaginal sex without condom in last 7 days with other partner (median)**	0 (0–1)	0 (0–1.5)	0.911

1 Fisher Exact test for categorical variables and Wilcoxon Mann Whitney test for continuous variables.

2 IQR = interquartile range.

3 Eligible: 49 Oral Contraceptions; 42 Injectable Contraceptions.

4 Ineligible: 1 Oral Contraceptions; 2 Injectable Contraceptions; 1 Implants.

### HIV incidence

A total of 283 women (73.1%) completed the study and remained uninfected; 22 women seroconverted (5.7%); 27 discontinued early (7.0%); and 55 were lost to follow-up (14.2%). We observed a total of 336.2 WY of follow-up, with a mean of 10.5 and median of 12.0 woman-months of observation. The overall retention rate (observed person-time divided by total possible person-time) was 80.0% for the entire follow-up period.

The 22 seroconversions yielded an overall prospective HIV incidence of 6.5 per 100 WY (95% CI: 4.1–9.9). The HIV incidence did not vary significantly by age (Table 2). The HIV incidence was steady through the entire 12-month observation period (Kaplan-Meier curve not shown).

**Table 2:** HIV incidence among women in prospective cohort, Beira, Mozambique.

	Value
**All women (18–35)**	
Had lab test result, N	387
Confirmed HIV seroconversion, N	22
Women-years of follow-up	336.2
Incidence (95% CI^1^)	6.5% (4.1, 9.9)
**Age 18–24**	
Had lab test result, N	309
Confirmed HIV seroconversion, N	19
Women-years of follow-up	266.5
Incidence (95% CI^1^)	7.1% (4.3, 11.1)
**Age 25–29**	
Had lab test result, N	55
Confirmed HIV seroconversion, N	3
Women-years of follow-up	49.6
Incidence (95% CI^1^)	6.0% (1.2, 17.7)
**Age 30+**	
Had lab test result, N	23
Confirmed HIV seroconversion, N	0
Women-years of follow-up	20.1
Incidence (95% CI^1^)	0.0% (0.0, 18.4)

1 Exact confidence interval.

### Factors associated with incident HIV

In bivariable analysis, factors that were associated with incident HIV infection (p≤0.05) were having had more than two sexual partners in the last month (HR 2.6; 95% CI: 1.1–6.0), having had a new sexual partner in the last month (HR 2.4; 95% CI: 1.0–5.6), number of vaginal sex acts without using condoms with other partners besides primary partner in the last 7 days (HR 1.6; 95% CI: 1.1–2.2) and using a form of contraception other than condoms, pills or injectables at baseline (HR 12.5; 95% CI: 1.6–99.1). Education, employment status, sex with an injecting drug user or having engaged in transactional sex in the last month, vaginal sex without using condom in the last seven days (with primary partner or other partner(s)), vaginal cleansing and vaginal insertions were not associated with incident HIV infection (Table 3).

**Table 3:** Factors associated with incident HIV infection among women in Beira, Mozambique.

	Bivariable Analysis		Multivariable Analysis	
	Unadjusted HR (95% CI)	p-value	HR (95% CI)	p-value
**Age**	0.9 (0.8, 1.1)	0.389	0.9 (0.8, 1.1)	0.307
**Education**				
> = Grade 10	1.5 (0.6, 3.5)	0.342	_	
<Grade 10	1			
**Employment**				
Yes (part-time or full-time)	0.8 (0.3, 2.1)	0.708	_	
No	1			
**Self-identified engagement in transactional sex**				
Yes	2.4 (0.9, 6.0)	0.073	1.5 (0.5, 4.2)	0.488
No	1		1	
**More than two sex partners last month**				
Yes	2.6 (1.1, 6.0)	0.024	1.9 (0.7, 4.9)	0.178
No	1		1	
**Had new sex partner(s) last month**				
Yes	2.4 (1.0, 5.6)	0.039	2.0 (0.8, 5.0)	0.136
No	1		1	
**Sex with IDU, MSM, or engaged in sex work in the last month**				
Yes	2.8 (0.4, 21.6)	0.321	_	
No	1			
**Frequency of vaginal sex without condom in last 7 days with primary partner**	0.9 (0.7, 1.3)	0.651	_	
**Frequency of vaginal sex without condom in last 7 days with other partner**	1.6 (1.1, 2.2)	0.006	1.7 (1.2, 2.5)	0.004
**Baseline contraception**				
Pills/injectables	1.0 (0.3, 3.2)	0.974	1.2 (0.4, 4.0)	0.763
Condoms	2.0 (0.8, 5.3)	0.143	2.1 (0.8, 5.6)	0.129
Other^1^	12.5 (1.6, 99.1)	0.017	25.3 (2.5, 253.5)	0.006
Nothing	1			
**Frequency of vaginal cleansing inside the vagina per month**				
31+	0.7 (0.1, 5.1)	0.710	_	
1–30	0.4 (0.0, 4.5)	0.461		
0	1			
**Frequency of vaginal insertions per month**				
1+	1.1 (0.4, 3.1)	0.787	_	
0	1			

1 Includes hormonal implant (1), IUD (1), and other (8).

In multivariable analysis, risk factors that remained statistically significant after controlling for other variables included: number of vaginal sex acts without using condoms (continuous variable) with other partners besides the primary partner in the last 7 days (HR 1.7; 95% CI: 1.2–2.5) and using a form of contraception other than condoms, pills or injectables vs. no method of contraception (HR 25.3; 95% CI: 2.5–253.5) (Table 3).

## Discussion

These are some of the first longitudinal HIV incidence data from Mozambique. We detected a high HIV incidence of 6.5 per 100 WY in this cohort of women at higher risk who underwent monthly risk-reduction counseling and received free condoms at all study visits.

These findings cannot be generalized to Beira as a whole or the larger Sofala Province population, since we targeted women at higher risk for HIV infection. Only 2% of women aged 15–49 years in Sofala Province reported two or more sexual partners in the past 12 months in the last national HIV surveillance survey (INSIDA, 2009). That eligibility criterion in our cohort led to a screening prevalence almost twice the overall provincial rate (32.6% [8]). The HIV prevalence may be stabilizing in Sofala Province, but that plateau can obscure a high HIV incidence in selected population segments, as has been reported in other regions of sub-Saharan Africa [9].

As in most African cohorts, our study participants reported a low prevalence (9.2%) of condom use for contraception, despite recent increases in condom availability in Mozambique. We also found a statistically significant association between HIV infection and the number of vaginal sex acts with other partners (besides the primary partner) without condom use in the last 7 days (HR 1.7). In Mozambique, weak condom use has been well documented and relates to issues of unequal relationships between women and men [10], [11]. Pinkerton and colleagues suggest that the number of unprotected sexual acts remains one of the best indicators of risk for STI transmission [12]. In Mozambique, unprotected sexual intercourse is highest among young people [4], [13]. Less than 15% of women and 20% of men reported using a condom at first intercourse in 2009 [4], underscoring the need to intensify condom programming in the young sexually active population.

In the multivariable analysis, we found that using a form of contraception at baseline besides a hormonal or condoms was associated with incident HIV infection. Given that there were only ten women in this group, this is almost certainly a chance finding.

Our study sample was skewed towards younger women (79% from 18–24 years) due to the heavy emphasis on recruitment in schools. The high HIV incidence in this group is consistent with findings from other African countries suggesting that women are most at risk of HIV in their teens and early twenties [14], compared to men who have the highest incidence in their early thirties. Retention in prospective research, however, can be attenuated when studying young cohorts [15].

Data on sexual behaviors were obtained via face-to-face interviews and self-reports. Participants could have over-reported socially acceptable behaviors and under-reported stigmatized behaviors. Furthermore, our study could not examine in depth a number of relevant factors such as mobility and migration, sexual relationships between people of different generations, and the details of transactional relationships [16], [17]. Transactional sex may be common in Mozambique, particularly in age-discrepant sexual encounters [18], although we did not observe an effect of inter-generational sex in our study.

Our study provides a rare estimate of the high HIV incidence among women with two or more sexual partners in Beira, Mozambique. Directly observed prospective HIV incidence remains the most credible method for ascertaining the state of an HIV epidemic, and an HIV incidence of around 3 per 100 person-years makes implementation of HIV prevention trials feasible [19]. Our study highlights that some groups in Beira far exceed that figure.

Most importantly, our findings have salient implications for HIV prevention planning in Beira, Mozambique. The high HIV incidence should incite vigorous HIV prevention efforts, particularly among women with multiple sexual partners. More research is needed to better understand the factors driving the HIV epidemic in Beira. The study reinforces the need for improved HIV testing and counseling and reproductive health services to facilitate early detection of HIV among young women.

Finally, the seriousness of the HIV epidemic among young people highlights the potential future burden on the health care system in Mozambique. Mozambique has 4 medical doctors and 39 nurses per 100,000 inhabitants, one of the lowest densities of medical personnel in the world [3]. Health authorities and non-governmental organizations should focus their efforts on strengthening HIV prevention services for young people and on the early detection of HIV to avoid further burdening the already over-whelmed health care infrastructure.
