# Correction: HIV Incidence in a Cohort of Women at Higher Risk in Beira, Mozambique: Prospective Study 2009–2012

**DOI:** 10.1371/journal.pone.0092530

**Published:** 2014-03-10

**Authors:** The PLOS ONE Staff

A funding organization and grant are incorrectly omitted from the Funding statement. The following sentence should be read with the Funding statement: "This study was supported in part by a Fogarty International Center AIDS International Training and Research Program grant, National Institutes of Health, to the University of Pittsburgh (D43TW001038).”
